# Families of transposable elements, population structure and the origin of species

**DOI:** 10.1186/1745-6150-6-44

**Published:** 2011-09-19

**Authors:** Jerzy Jurka, Weidong Bao, Kenji K Kojima

**Affiliations:** 1 Genetic Information Research Institute, 1925 Landings Drive, Mountain View, CA 94043, USA

## Abstract

**Background:**

Eukaryotic genomes harbor diverse families of repetitive DNA derived from transposable elements (TEs) that are able to replicate and insert into genomic DNA. The biological role of TEs remains unclear, although they have profound mutagenic impact on eukaryotic genomes and the origin of repetitive families often correlates with speciation events. We present a new hypothesis to explain the observed correlations based on classical concepts of population genetics.

**Presentation of the hypothesis:**

The main thesis presented in this paper is that the TE-derived repetitive families originate primarily by genetic drift in small populations derived mostly by subdivisions of large populations into subpopulations. We outline the potential impact of the emerging repetitive families on genetic diversification of different subpopulations, and discuss implications of such diversification for the origin of new species.

**Testing the hypothesis:**

Several testable predictions of the hypothesis are examined. First, we focus on the prediction that the number of diverse families of TEs fixed in a representative genome of a particular species positively correlates with the cumulative number of subpopulations (demes) in the historical metapopulation from which the species has emerged. Furthermore, we present evidence indicating that human AluYa5 and AluYb8 families might have originated in separate proto-human subpopulations. We also revisit prior evidence linking the origin of repetitive families to mammalian phylogeny and present additional evidence linking repetitive families to speciation based on mammalian taxonomy. Finally, we discuss evidence that mammalian orders represented by the largest numbers of species may be subject to relatively recent population subdivisions and speciation events.

**Implications of the hypothesis:**

The hypothesis implies that subdivision of a population into small subpopulations is the major step in the origin of new families of TEs as well as of new species. The origin of new subpopulations is likely to be driven by the availability of new biological niches, consistent with the hypothesis of punctuated equilibria. The hypothesis also has implications for the ongoing debate on the role of genetic drift in genome evolution.

**Reviewers:**

This article was reviewed by Eugene Koonin, Juergen Brosius and I. King Jordan.

## Background

Eukaryotic genomes contain multiple copies of TEs historically known as "interspersed repetitive DNA." This repetitive DNA originates from different classes of TEs, multiplying and integrating themselves or other DNA in the host genomes at different evolutionary periods. TEs play an intrinsic role in genome evolution [1-5] and are even viewed by some as major drivers of genome evolution including speciation events [6-9]. All interspersed repeats tend to be composed of distinct families in terms of their age and DNA sequence characteristics, as was first documented in the case of human Alu elements [10-15]. It is generally accepted that most, if not all, large mammalian families of TEs originate from a small number of actively expressed copies called "source", "founder" or "master" genes and produce discrete families of "repetitive elements".

Sequence studies point to a large diversity among TE families originating in different species. For example, mammals tend to harbor a small number of large families with few recently active elements whereas plants, insects and fish frequently harbor numerous small families composed of very young TEs [16]. Furthermore, many families of TEs are species-specific even in species from the same lineage. Biological properties of TEs and their interactions with the host molecular environment do not satisfactorily explain these observed patterns.

In this paper we propose that the observed species-specific differences between families of TEs are determined, at least to a large extent, by the population structure of the host. Fixation of TEs can take place in small populations by genetic drift [17-20], and small population sizes might have played a major role in fixation of non-adaptive DNA in eukaryotes, including TEs [21-23]. Recently a link between accumulation of TEs and small population sizes was hypothesized in the context of speciation [24]. Here we further explore this link to address the observed lineage-specific properties of repetitive families and their coincidence with speciation events.

A large population with a heterogeneous pool of active TEs can subdivide into small subpopulations (demes), each carrying a random set of active elements. If the subpopulations are small enough and gene flow among them slow enough, they will be subject to genetic drift [25]. Some of the subpopulations may eventually evolve into independent species, each with unique assortment of repetitive families derived from the founding set of active elements. This is known as the "founder effect" that was first fully articulated by Ernst Mayr in 1952 (see [26]). Those demes that do not evolve into a new species may go extinct or exchange their genetic material within the population and contribute to its genetic diversity. If the hypothesis is correct, then studies of repetitive families can be of fundamental importance for understanding the relationship between population structure and speciation.

## Presentation of the hypothesis

### The carrier subpopulation (CASP) hypothesis

We assume that most eukaryotic populations include individuals that carry a variety of active TEs (source genes), not fully suppressed by the silencing mechanisms. Active TEs that survive the silencing are likely to be outliers in terms of their mosaic structure (e.g. new SINE elements), or sequence divergence. We also assume that the fixation of repetitive families takes place primarily in small populations by genetic drift and that the transposed copies of TEs are neutral or slightly harmful. The number of TEs fixed under relaxed selection in small populations is determined primarily by the rate of transposition and the time during which the elements were active [27-29].

**Figure 1:**
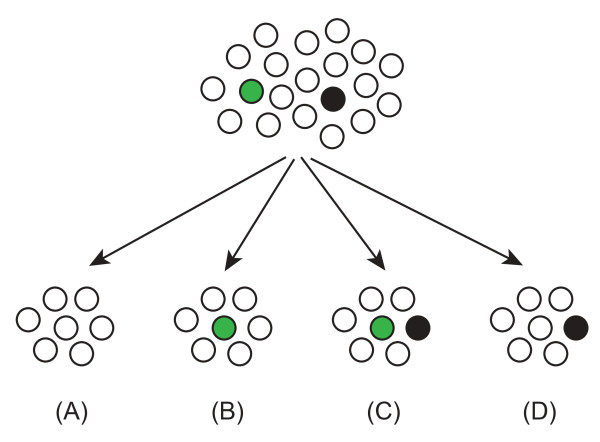
**Schematic subdivision of a population into subpopulations**. Colored circles represent active TEs (source genes).

Figure 1 schematically illustrates a hypothetical splitting of a large population, with a pool of active TEs, into small subpopulations that inherit either some of the active TEs (Figure 1B, C, D), or none (Figure 1A). The inherited active elements can produce new families of repetitive elements primarily due to fixation by genetic drift in small subpopulations. If two or more different types of TEs are active in a small subpopulation (Figure 1C), they may generate multiple families of similar age due to parallel fixation by genetic drift.

Most subpopulations will remain connected to their metapopulations through migrations of their genetic material (gene flow), and the distribution of different families of TEs in metapopulations is likely to be determined by a balance between genetic drift and migrations, analogously to the distribution of neutral genes [30]. Over time, highly structured metapopulations are expected to accumulate unique sets of diverse families of TEs due to local fixations in subpopulations, often exposed to local environmental challenges, followed by migrations [31-33]. This diversity will eventually be reflected by the number of different families fixed in the genomic DNA of the surviving species. The hypothesis implies that highly subdivided populations will harbor multiple families of diverse TEs and vice versa. The size of each family is expected to positively correlate with the rate of transposition and to negatively correlate with the rate of genetic flow between different subpopulations.

### Carrier subpopulations and the origin of species

The potential involvement of TEs in speciation has been discussed since the early eighties based on the contributions of Barbara McClintock (reviewed in [34]), and the discovery of hybrid dysgenesis [35,36]. The common thread of these early hypotheses was that bursts of TEs could contribute to reproductive isolation between some populations, which eventually can evolve into new species. However, over time the general appeal of those ideas has diminished as it became clear that hybrid dysgenesis may not be sufficiently common to support a more general involvement of TEs and their contribution to reproductive isolation remained elusive [37,38]. Nevertheless, more recent studies of interspecific hybridization and its role in activation of TEs [39-42] bring a renewed interest in the classical ideas of speciation and the involvement of TEs [43,44]. The ubiquitous amplification of TEs by genetic drift in subdivided populations may provide a new framework to pursue those classical ideas.

The CASP hypothesis proposes that the population subdivision triggers fixation of repetitive families by genetic drift. Therefore, new repetitive families are likely to coincide with the origin of a new species from small subpopulations whether or not TEs contribute to the process of speciation. At the same time, accumulation of TEs in different subpopulations may eventually help them to "drift apart" due to their mutagenic impact driven by the rate of transposition, which can increase the probability of reproductive isolation due to genetic diversification. Such mechanistic scenarios were already explored in the early eighties [45], but the general role of genetic drift in amplification of repetitive families was not clearly recognized at the time. The population subdivision and the resulting diversification of local subpopulations will also inevitably lead to occasional formation of hybrids combining ancestral mutations and accumulating new ones generated by active TEs introduced during crossbreeding. From this perspective, the emergence of a new species is a process rather than an event and it is likely to involve multiple factors [46], including the TE-mediated diversification of different subpopulations.

The TE-mediated diversification is most likely to cause productive speciation if TEs can expand to large families in a relatively short time and increase the chances of reproductive isolation. Probably only a few subpopulations can survive large bursts of mutations and only a few active TEs can reach such bursts of activity. Therefore, large repetitive families are probably derived from a small number of the most active TEs, which is consistent with the "master gene" hypothesis [15]. Slowly replicating TEs are probably less likely to affect speciation. They are also less destructive and, as such, less frequently suppressed by the silencing mechanisms. Therefore, they are more likely to be represented by multiple active copies as proposed by the "transposon model" [47,48].

The rapid TE-mediated diversification is likely to be evolutionarily significant in populations that cannot easily subdivide into isolated subpopulations for any extended period of time. Highly dispersible populations are less likely to produce species with large species-specific repetitive families. Furthermore, the critical mass of mutations that contribute to the effective separation of different subpopulations is expected to positively correlate with genome size. As new species emerge, and their populations continue to grow, the rate of fixation of new TEs by genetic drift is likely to decline. It can be revived again by new cycles of subdivisions producing small subpopulations and new families of TEs.

## Testing the hypothesis

### Diverse families of TEs and the population structure

Table 1 illustrates diversity of TEs in vertebrates, insects and plants [16] for selected, well annotated species from Repbase [49,50]. The diversity is defined as the number of different TE families per species (listed in columns 2 and 3), irrespective of their biological classification. To simplify the analysis, we do not distinguish between families and subfamilies of TEs [49-51]. Also, throughout this paper we focus primarily on the analysis of relatively young families, which are better preserved than the older ones and can be more reliably identified and classified. As is shown in the Table, the numbers of diverse families in two different mosquito species (*A. gambiae *and *A. aegypti*) differ by a factor of magnitude. Analogous, although less dramatic differences in the diversity of TEs can be seen in vertebrates and plants. Among the four plants listed, the most "primitive" ones (*S. moellendorffii and P. patens*) have the lowest numbers of different families of TEs.

**Table 1:** Diversity of TEs in selected species

Species	No. Families < 1% divergent	No. Families < 5% divergent
*Danio rerio*	124	217
Mammals (range)	0-23	5-113
*Xenopus tropicalis*	26	91
*Anopheles gambiae*	50	115
*Aedes aegypti*	561	1093
*Drosophila melanogaster*	19	49
*Arabidopsis thaliana*	52	199
*Zea mays*	149	265
*Selaginella moellendorffii*	11	24
*Physcomitrella patens*	4	8

Diversity of TEs is defined as the number of different families per genome. DNA sequence divergence for individual families is defined as a percentage of base substitutions either relative to their consensus sequences or between LTRs in the case of LTR retrotransposon families.

The CASP hypothesis implies that the number of diverse families of TEs fixed in a genome of an individual representing a particular species will positively correlate with the cumulative number of subpopulations (demes) in the historical metapopulation from which the species has emerged. Based on the hypothesis we predict that the population of *A. aegypti *has been more subdivided in its recent evolutionary history than that of *A. gambiae*. This is consistent with the apparent adaptability of *A. aegypti *as it spread to new geographical locations in recent history [52]. In plants, more primitive species (*S. moellendorffii and P. patens*) probably generated fewer subpopulations in their recent evolutionary history than the more modern ones (*A. thaliana, Z. mays*). This may be due to lack of niches that could be successfully invaded by new subpopulations due to competition from modern plant species. Invasion of new niches may also be difficult for highly specialized organisms, including colonial insects and parasites, some of which are known to have no active TEs [53].

**Figure 2:**
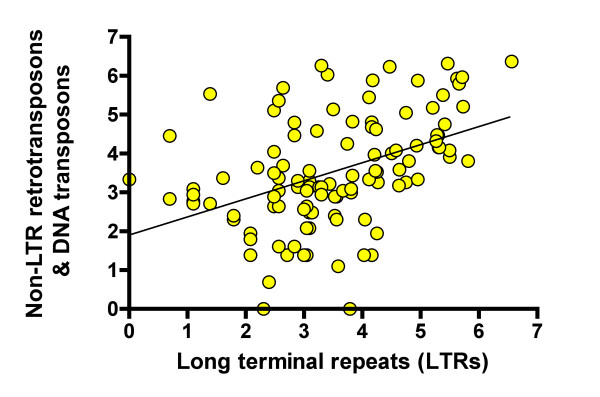
**Correlation between unrelated TE families in diverse groups of species**. Vertical axis: natural logarithms of cumulative numbers of non-LTR retrotransposon and DNA transposon families. Horizontal axis: natural logarithms of cumulative numbers of LTR retrotransposon families.

Active TEs may develop complex relationships with each other and with the host environment [16,54], which may lead to a competitive relationship and negative correlation between families derived from biologically unrelated TEs such as LTR and non-LTR retrotransposons. On the other hand, the CASP hypothesis predicts positive correlation between the total number of diverse families and the number of subpopulations in metapopulations. This implies that any two subsets of biologically unrelated families should also positively correlate with each other as they both correlate with the number of subpopulations. To test this prediction, we analyzed correlation between the overall numbers of families of long terminal repeats (LTRs) and all the remaining families derived from non-LTR retrotransposons and DNA transposons, for 111 groups of species represented in Repbase [49,50]. The species were combined into groups based on the first part of their binomial names. For example, all Drosophilidae (*D. melanogaster, D. mojavensis, D. pseudoobscura*, etc.) were combined into a single group. In the next step, we calculated the total number of different LTRs deposited in Repbase, and the analogous combined number of all the remaining TEs for the same group of species. Each sequence deposited in Repbase represents a single family of TEs [51]. In this way we obtained 111 pairs of numbers representing the total counts of LTR families and of the remaining TE families for each group of species, excluding mammals, which were analyzed separately. We calculated linear correlation between logarithmic values of the corresponding family counts due to a substantial positive skew in their distribution (Figure 2: r = 0.43; P < 0.0001). In a separate analysis, a significant positive correlation was also found between analogous sets of mammalian repetitive families less than 15% divergent from their consensus sequences (r = 0.74; P < 0.0001). However, the significance was marginal for families < 5% divergent from their consensus sequences due to relative underrepresentation of young families in mammals.

### Parallel fixation of multiple TE families in a subpopulation

A small subpopulation can "incubate" multiple repetitive families fixed by genetic drift, provided that it carries multiple active TEs (Figure 1C). This may lead to the corresponding species-specific assortments of diverse repetitive families if the subpopulation becomes the founder populations for new species. The parallel fixation model also predicts that repetitive families generated by TEs in small populations are likely to be of similar age and are likely to be accompanied by contemporary fixations of unrelated mutations such as base substitutions, satellite amplifications, chromosomal rearrangements etc.

Currently, the most detailed comparative data on multiple repetitive families of TEs are available for mammalian species (Table 2). Most young families of TEs listed in Table 2 (< 1% divergent from their respective consensus sequences), are species-specific, i.e. they are closer to each other within species than between species. In this respect they most likely reflect a stochastic assortment of diverse active TEs from which they were derived. Mammalian TEs are derived from two major types of TEs using different mechanisms of reproduction: L1 retrotransposons (LINEs, SINEs) and endogenous retroviruses. However, in bat (*Myotis lucifugus*), these two groups are accompanied by at least two different types of DNA transposons (*hAT, Mariner*). Some of them (e.g. SPIN_NA_1_Et) were probably introduced by a horizontal transfer [55], but they all appear to have expanded into repetitive families in a similar period of time. According to one variant of the CASP hypothesis, this could be due to favourable conditions for fixation by genetic drift in a small subpopulation ancestral to at least one species of bats. The same logic applies to older likely horizontal transfers in mammals such as *Vingi *TEs in hedgehogs [56] or *RTE *TEs in ruminants [57], except that families derived from these TEs are shared by multiple species descending from the ancestral population in which the horizontal transfer and fixation originally took place.

**Table 2:** Diversity of TEs in mammalian species.

Species (genome size)	< 1% divergence			< 5% divergence	
	
	Repbase ID names (genomic copy number in parentheses)	No. Families	Total Count	No. Families	Total Count
Human (2.87GB)	AluYa5(2696), AluYb8(1499), AluY(215), AluYc1(433), L1HS(332), AluYg6(242), AluYb9(213), AluYd8(122), AluYa8(40), LTR5_Hs(38), AluYh9(18), HERVK(6).	12	5981	48	33613
Chimpanzee (2.90GB)	AluY(150), AluYc1(1320), PTERV1c_LTR(80), CERV1_LTR(53), L1Pt(24), AluYa5(11), AluYb8(7).	8	1799	50	23832
Rhesus macaque (2.64GB)	AluMacYb2(1122), AluMacYb4(1047), AluMacYa3(364), L1_RS1_5end(69), MacERVK2_LTR1a(52), MacERV1_LTR4a(40), MacERV1_LTR3a(23), MacERV3_LTR2(22), MacERV4_LTR1a(22), MacERV4_LTR4(20), MacERV3_LTR3(14), MacERV2_LTR1(11), MacERVK2_LTR1c(11), MacERV1_LTR4d(9), MacERV2_LTR2a(8).	15	2834	67	70319
Marmoset (2.76GB)	HAL1-1C_Cja(32).	1	32	11	1299
Tarsier (2.77GB)	LTR10_TS(11), LTR13_TS(7).	2	18	23	7705
Mouse lemur (1.85GB)	LTR3_Mim(49), ERV1-Mim_LTR(23), LTR10_Mim(22), LTR1_Mim(22), ERV2N1-Mim_LTR(14), LTR10B_Mim(8), LTR14_Mim(5).	7	143	26	6066
Bushbaby (1.97GB)	GarnAlu1(80), LTR8_OG(21).	2	101	12	18970
Mouse (2.62GB)	L1_MM(1244), RLTRETN_MM(154), IAPLTR1_Mm_LTR(65), MMERGLN_LTR(59), IAPLTR1a_MM(58), RLTR1IAP_MM(54), RLTR6B_Mm(39), B1_Mm(34), B2_Mm1a(30), IAPEY4_LTR(30), LTRIS_Mm(26), B1_Mus2(23), ERVB7_1-I_MM(22), ORR1A0(20), ERVB7_1-LTR_MM(11), IAPLTR1a_I_MM(11), ERVB4_1B-LTR_MM(10), MTA_Mm_LTR(10), MMERVK10C(8), IAPLTR3(6), RLTR13B2(6), L1Md_Gf_5end(5), LTRIS3(5).	23	1930	113	72236
Rat (2.48GB)	ID_Rn1(4510), B2_Rn(349), L1_RN(212), NICER2_Rn(113), ID_Rn2(107), RNLTR12(73), B2_Rn1(61), RNLTR19A(61), RNLTR8C(54), B2_RnY(50), NICER1B_Rn_LTR(37), RNIAP1aLTR(26), RNIAP1bLTR(18), RNLTR8C2(16), RNNICER3_I(15), B2_Rat2(12), RNLTR12-int(11), RNLTR2a(9), NICER3A_Rn(8), B2_Rn2(7), ERVB5_3-LTR_RN(6).	21	5755	92	67356
Kangaroo rat (1.84GB)	DIPODE1(126), BC1_Dor(115), DERV2a_LTR(32), LTR13B_Dor(18), L1-1_Dor(14), ERV2-Dor_LTR(9).	6	314	21	54065
Guinea pig (2.66GB)	CAVID(392), L1-1C_Cpo(377), L1-1D_Cpo(139), HAL1-1H_Cpo(106), HAL1-3D_Cpo(54), HAL1-1G_Cpo(50), HAL1-1E_Cpo(37), ERV1NA-CPo_LTR(36), 5S_CPo(15), ERV1A1-CPo_LTR(9).	10	1215	39	38173
Squirrel (1.91GB)	LTR3_Str(65), ERV2-1_STr-LTR(40), LTR27_Str(19), STRID3(9), LTR8_Str(7).	5	140	47	13754
Rabbit (2.08GB)	LTR26_OC(24), LTR10_OC(23), ERVH_OC_LTR(5).	3	52	36	47855
Pika (1.92GB)	CSINE3_OP(210), ERV2-1N-OP_LTR(48), LTR2B1_Opr(40), ERV2-1-LTR_Opr(31), ERV2-4_Opr-LTR(22), LTR10_Opr(14), LTR30_Opr(10), LTR7_Opr(10), ERV1-1_OPr-LTR(8), ERV2-2_Opr-LTR(8).	10	401	54	14259
Cow (2.73GB)	ERV2-1-LTR_BT(25), BTLTR1B(14), L1-BT(7).	3	46	31	63422
Alpaca (1.92GB)		0	0	10	420
Dog (2.38GB)	L1-Y_CF(354), SINEC2A1_CF(79), CfERVF1_LTR(18), SINEC2A2_CF(11).	4	462	14	81969
Cat (1.64GB)	SINEC_Fc(2312), ERV1-2_FCa-LTR(36), ERV1-3_FCa-LTR(24).	3	2372	16	59515
Horse (2.42GB)	ERE1(532), L1-1_EC(61)	2	593	20	19683
Megabat (1.83GB)	ERV1-1_PVa-LTR(102), ERV2-2_PVa-LTR(21), ERV2-3_PVa-LTR(12), LTR15_PVa(5).	4	140	16	1924
Bat (1.67GB)	nhAT6_ML(1428), nhAT4a_ML(975), nhAT2_ML(823), nhAT1_ML(310), nhAT5b_ML(295), hAT-2N2_ML(227), nhAT3_ML(167), nhAT5a_ML(13), LTR30A_ML(9), ERV1X1-LTR_ML(7), ERV2X1-LTR_ML(7), SPIN_NA_1_Et(6), Ves(6), LTR27D2_ML(5), MARIN1_ML(5).	15	4283	55	99014
Hedgehog (2.13GB)	Vingi-1N3_EE(311), LTR4_EE(54), LTR1C_EE(38), ERV2-4_EE-LTR(28), (13), ERV2-1_EE-LTR(11), LTR26_EE(8), ERV2-3_EE-LTR(7), LTR13_EE(5).	9	475	63	14890
Shrew (1.83GB)	LTR6_Sar(45), LTR1_Sar(22), LTR2_Sar(22), LTR7_Sar(10), LTR8_Sar(8), LTR18C_Sar(5).	6	112	25	2803
Elephant (2.30GB)		0	0	7	1052
Tenrec (2.11GB)		0	0	5	4783
Hyrax (2.41GB)	ERV2-6_Pca-LTR(166), ERV2-3_Pca-LTR(130), ERV2-1-Pca_LTR(56), MLT1Z_Pca(16), ERV1-2_PCa-LTR(8), LTR15B_Pca(7).	6	383	40	9945
Armadillo (2.15GB)		0	0	6	5950
Sloth (2.12GB)	ERV2N1_CHo-LTR(402), LTR3_Cho(142), ERV2N1B_CHo-LTR(6), LTR5A_Cho(6).	4	556	23	3709
Opossum (3.34GB)	L1-1_MD(953), SINE-1_MD(193), ERV2_MD_I(109), ERV2_MD_LTR(70), L1A-2_MD(42), MARINERNA6_MD(10), LTR32_MD(9), ERV1_MD_I(7).	8	1393	70	46497
Wallaby (2.54GB)	MERVK1B_LTR(10), MERVK1D_LTR(5).	2	15	7	9514
Platypus (1.84GB)		0	0	10	214

Each family is composed of at least five elements per genome. Column 1: common species name and the size of the sequence data. Column 2: Repbase ID names of families with < 1% of divergence from their consensus sequences. Columns 3 and 5: the number of different families (< 1% and < 5% divergent from their respective consensus sequences). Columns 4 and 6: the corresponding total combined numbers per genome (total counts). Scientific names of species used are as follows: human, *Homo sapiens*; chimpanzee, *Pan troglodytes*; Rhesus macaque, *Macaca mulatta*; marmoset, *Callithrix jacchus*; tarsier, *Tarsius syrichta*; mouse lemur, *Microcebus murinus*; bushbaby, *Otolemur garnettii*; mouse, *Mus musculus*; rat, *Rattus norvegicus*; kangaroo rat, *Dipodomys ordii*; Guinea pig, *Cavia porcellus*; squirrel, *Spermophilus tridecemlineatus*; rabbit, *Oryctolagus cuniculus*; pika, *Ochotona princeps*; cow, *Bos taurus*; alpaca, *Lama pacos*; dog, *Canis lupus familiaris*; cat, *Felis catus*; horse, *Equus caballus*; megabat, *Pteropus vampyrus*; bat, *Myotis lucifugus*; hedgehog, *Erinaceus europaeus*; shrew, *Sorex araneus*; elephant, *Loxodonta africana*; tenrec, *Echinops telfairi*; hyrax, *Procavia capensis*; armadillo, *Dasypus novemcinctus*; sloth, *Choloepus hoffmanni*; opossum, *Monodelphis domestica*; wallaby, *Macropus eugenii*; platypus, *Ornithorhynchus anatinus*.

### Origin of multiple families by crossbreeding

The human and chimpanzee genomes share some Alu subfamilies such as AluYc1 and a small number of AluYa5- and AluYb8-like copies, but most young families present in humans are absent from chimpanzees (e.g. L1HS, AluYg6, AluYb9, AluYd8, AluYa8, LTR5_Hs, AluYh9, HERVK). One possibility is that that they were fixed in parallel in a single proto-human population after its separation from the chimpanzee lineage. Alternatively, some of them might have originated in separate subpopulations and merged by subsequent crossbreeding. Natural hybridization in mammalian populations has recently been demonstrated in bats as a rare mechanism for the origin of new species [58].

The possibility of hybridization between individuals from diverse subpopulations is intrinsically associated with population subdivision and analysis of species-specific families of TEs may help us to understand whether or not the hybridization contributed to the origin of a particular species. Here we analyze the possibility that AluYa5 and AluYb8 originated in separate proto-human populations. The ratios of chromosomal densities of AluYa5 elements are consistent with the model of paternal transmission, i.e. inheritance of active Alu elements through male germ line only [59,60]. The density ratio of AluYb8 elements on chromosome X relative to autosomes (~0.71) is comparable to that for AluYa5 (~0.72) and is consistent with paternal transmission, which predicts the ratio to be equal to 0.67 (2/3). However, the ratios of AluYb8 densities on chromosome Y relative to autosomes (~0.9) and chromosome X (~1.3) are much lower than the corresponding values for AluYa5 elements (~1.94 and ~2.52), and the values predicted by the paternal transmission model (2.0 and 3.0, respectively). The overall number of AluYb8 elements on chromosome Y is lower by at least 40% than expected by extrapolation from the observed ratios of AluYa5 chromosomal densities. Closer inspection indicates an underrepresentation of older AluYb8 elements on chromosome Y (not shown).

The underrepresentation of older elements among young Alu families on chromosome Y was indirectly noted before [61]. It was then proposed that Alu elements were probably removed from the male sex chromosome over time. Alternatively, they might have been missing from the beginning if some young Alu families were introduced to the human genome by crossbreeding with another, now extinct, proto-human population. According to this scenario, older AluYb8 elements that were already fixed in the "Yb8 population" could be passed to the hybrid population by crossbreeding and spread over the population by homologous recombination between chromosomes except of chromosome Y, which does not recombine outside its pseudoautosomal regions. It is proposed that the surviving chromosome in the hybrid population carried the original AluYa5 elements but acquired AluYb8 elements by retroposition after the crossbreeding took place. In other words, the surviving human chromosome Y might have been exposed to a shorter "bombardment" [62] by the AluYb8 elements than by the indigenous AluYa5 elements and this is why an older fraction of AluYb8 elements is missing. This scenario may apply to other human-specific Alu families, and beyond.

### Relationship between families of TEs and speciation

The presence of lineage-specific amplification of SINE elements was first observed over a decade ago [63], and successfully used in biological systematics, particularly in mammals [64-73]. According to the hypothesis, large lineage- or species-specific bursts of repetitive families [56,74-79] are consistent with their involvement in rapid diversification of subpopulations that could lead to speciation. Other studies of the human genomic fossil record revealed temporal correlation between outbursts of fixation of retropseudogenes and different speciation events, based on analysis of retropseudogenes in the primate lineage [80]. The observed outbursts of retropseudogenes are likely to be caused by the high activity of L1 elements that are also responsible for large species-specific outbursts of SINE families.

**Figure 3:**
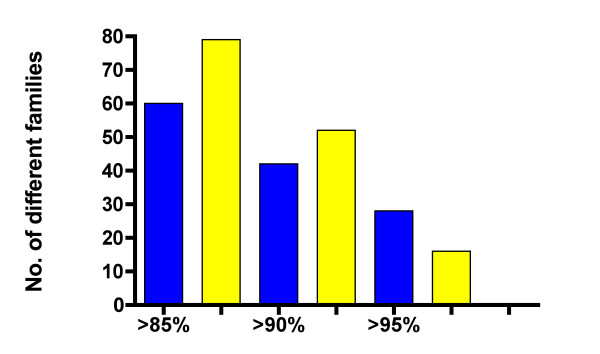
**Cumulative numbers of TE families from the rabbit and pika genomes**. The bars indicate numbers of TE families > 85%, > 90% and > 95% identical to their respective consensus sequences in the genomes of rabbit (yellow) and pika (blue).

There is also an apparent correlation between activity of TEs and mammalian taxonomies. To illustrate the pattern, we focus on sample analysis of "young" families in mammals representing lineages with different taxonomic structure. Table 2 (column 3) lists the numbers of different families, less than 1% divergent from their respective consensus sequences, for most mammalian genomes sequenced to date. For example, the pika (*Ochotona princeps*) genome carries 54 families < 5% divergent from their consensus sequences (Table 2, column 5), of which 10 are relatively young (< 1% divergent). The corresponding numbers of families for rabbit (*Oryctolagus cuniculus*) are 36 and 3. *O. princeps *belongs to genus *Ochotona *represented by ~30 species, and *O. cuniculus *is the only species in genus *Oryctolagus*. However, *O. cuniculus *belongs to the *Leporidae *family that includes several different genera of rabbits and hares representing around 50 species, whereas all 30 species of pika belong to a single genus only. This suggests earlier major speciation events in rabbits, associated with amplification of older families of TEs, consistent with phylogenetic analysis based on activities of SINE elements [70]. Figure 3 shows the number of different families of TEs in rabbit (yellow) and in pika (blue) for three different time intervals measured by sequence similarity to consensus. Older families with elements > 85% and > 90% identical to consensus are more abundant in rabbit than in pika. Conversely, families > 95% identical to consensus are more numerous in pika than in rabbit, consistent with more recent speciation events in pika. In general, higher taxonomic levels reflect older speciation events and are expected to be associated with fixation of older families of TEs in more ancient subpopulations. This is in line with the view that species are implicitly identified by reference to particular historical populations [81].

The number of surviving species in a lineage reflects the rate of speciation less extinctions that occur over time, i.e. more ancient speciation events are likely to leave fewer surviving species in the lineage than the more recent ones. According to the CASP hypothesis, the relatively large number of species observed in some orders may reflect evolutionarily recent population subdivisions and speciation events associated with recent amplification of TEs by genetic drift rather than the TE-triggered "fecundity" of the lineages [8,9]. For example, mammalian species with the highest numbers of young families in Table 2, such as mouse (23 families), rat (21), bat (15), Rhesus macaque (15) and human (12), represent the largest mammalian orders of Rodentia, Chiroptera and Primates. On the other end of the spectrum are species representing relatively species-poor lineages such as alpaca, elephant, tenrec, armadillo and platypus. They do not harbor any young families of TEs (< 1% divergent from their consensus sequences), which, according to the hypothesis indicates limited population subdivisions and speciation in their recent evolutionary history. However, phylogenetic analyses clearly indicate more ancient speciation events associated with older families of TEs.

## Implications of the hypothesis

Recently, it has been proposed that TEs constitute the main engine of the so called "genomic drive", which according to the hypothesis can explain various aspects of speciation [8,9]. If the CASP hypothesis is correct, then major speciation events are driven not by "periodic infestations" by TEs [8] but by subdivisions of populations into small subpopulations, some of which may become founder populations for new species. It also proposes that small populations resulting from the subdivisions are primary incubators of new repetitive families derived from TEs, which are fixed by genetic drift. Fundamentally, TEs should be viewed more as drifters accompanying population subdivision rather than the drivers of speciation. The accumulation of TEs by genetic drift does not rule out their involvement in various evolutionary processes, including speciation. In fact, the intrinsic association between population subdivision and fixation of TEs proposed by the CASP hypothesis makes such involvement inevitable. TEs can act as mutator genes to bring opportunistic bursts of mutations at critical evolutionary junctions leading to new species [24,82]. Many bursts of mutations caused by the most active TEs are likely to have catastrophic consequences and the resulting multiple extinctions of small populations are expected to accompany speciation events. However, the surviving subpopulations are prime candidates for founder populations for new species. Massive injection of new repetitive elements is likely to be followed by periods of genetic instabilities with multiple evolutionary consequences for the surviving populations. Some repetitive elements subject to purifying selection over time [83] might have been recruited during such periods.

The origin of repetitive families is associated with historical population structures that existed during speciation events. The layers of repetitive elements added to genomic DNA by genetic drift can account for fluctuations in the amounts of the non-coding genomic DNA in different species. This is consistent with some earlier empirical observations [84,85] and with the original hypothesis by Lynch and Conery [21], linking effective population size (*N_e_*) to genome size and proposing a central role of non-adaptive processes in the evolution of genome architecture. The hypothesis attracted substantial interest due to its potential explanatory power [86], but it continues to be debated due to the apparent lack of significant correlation between *N_e _*and genome size in the context of phylogenetically restricted models [87-89]. The CASP hypothesis predicts a correlation between *N_e _*of historical populations and the corresponding layers of repetitive DNA deposited in the genome rather than the overall correlation between the *N_e _*of contemporary populations and the total genome size. Therefore, the fixation of TEs by genetic drift cannot be separated from their phylogenetic history and must be analyzed in the context of historical populations.

The origin of new subpopulations can be fueled by the availability of new ecological niches due to geographical factors or mass extinctions. New niches can also be opened by major evolutionary breakthroughs such as the emergence of echolocation in bats [90] or of the adaptive immune system in vertebrates [91]. The hypothesis implies that punctuated equilibria [92] reflect rare evolutionary breakthroughs during the history of life that lead to exploration of new niches accompanied by subdivisions of the population and speciation. Consequently, the outbursts of discrete families of different age preserved in mammalian genomes are likely to reflect major invasions of new niches by the ancestral mammalian populations. In short, this points to a "niche-driven speciation hypothesis" rather than the "genomic drive hypothesis."

The presence of young families of TEs in the genomic fossil record can be a powerful indicator of the evolutionarily recent or ongoing subdivisions in the population that underlie speciation events. However, the lack of young families in a genome is less informative because it may be due to a random loss of active source genes in the founder populations. Even organisms losing important active TEs such as LINE1 elements [93] can represent structured populations in the process of active adaptation to new niches. This may suggest that the mutagenesis by TEs is not essential for speciation, at least in the short run. Alternatively, other types of TEs or unrelated mutations such as chromosomal rearrangements, satellite amplifications or nucleotide substitutions may compensate for the loss of the mutagenic impact of the LINE1 family. The long-term survival of TEs in most eukaryotic lineages is a strong argument in support of their evolutionary importance.

In summary, families of TEs deposited in genomic DNA are likely to reflect structural changes in historical populations caused by recurrent waves of colonization, drifting apart, extinctions and crossbreeding which can be studied in contemporary populations [58,94-97]. A prolonged lack of any significant fixation of TEs in a population may indicate that the population is locked in a constrained niche with little room for further colonization and population subdivision. Over time, the reduced activity of TEs may translate to smaller genome sizes unless it is offset by DNA amplifications unrelated to expansion of TEs.

During the final stages of the preparation of this paper we became aware of a recently proposed "frozen plasticity theory" [98], which also emphasizes a role of population subdivision in the process of speciation and links population subdivisions to punctuated equilibria based on evidence unrelated to TEs. The frozen plasticity theory sparked a vigorous debate [98], emphasizing the need for systematic studies and new evidence that could further the fundamental understanding of speciation. If families of repetitive elements are associated with speciation as proposed by our hypothesis, this could open a goldmine of genomic fossil data directly relevant in this debate.

## List of abbreviations used

TE(s): transposable element(s); SINE: short interspersed element; LINE: long interspersed element; CASP: carrier subpopulation; LTR: long terminal repeat; *N_e_*: effective population size.

## Competing interests

The authors declare that they have no competing interests.

## Authors' contributions

JJ developed the hypothesis and wrote the manuscript. WB and KKK contributed to data preparation, analysis and final revisions of the manuscript. All authors read and approved the paper.

## Reviewers' comments

### Reviewer 1

Eugene V. Koonin, NCBI, NLM, NIH, Bethesda, MD 20894, USA

Jurka and colleagues address the connections between transposable elements propagation and speciation of the host organisms. The principal message of the article is very simple: TEs might not be a causative factor in speciation. Rather, waves of TE propagation are thought to coincide with speciation because both phenomena are triggered by the division of large populations into small sub-populations (demes) in which drift is important.

I find this hypothesis to be perfectly sensible and actually a better null hypothesis than the scenario with the causal role of TEs in speciation. The hypothesis presented in this paper is fully in line with Lynch's population-genetic hypothesis on non-adaptive evolution of complexity, and I am surprised that the paper makes no reference to this theory (Lynch 2007; Lynch 2007).

**Response**: *These important references were accidentally lost from the Backgroud section during formatting. They were restored and they are also discussed in the Implications section.*

I further wonder whether the argument presented in this paper could be coached in quantitative terms by using the data on the abundance of TEs in different mammalian genomes to estimate just how small those subpopulations should have been to sustain the observed level of TE propagation. This estimate would be analogous to Lynch's and my own estimates of the effective population size required to maintain intron-rich genes (Lynch 2007; Koonin 2009). I think such estimate would make the story more interesting and compelling.

**Response**: *Such estimate may be more complex as the CASP hypothesis is centered on genetic drift in the context of historical population structures associated with speciation events. This also has implications for the ongoing debate on the phylogenetic context of genome evolution (see the discussion section and refs. 87-89). We believe that any specific quantitative models deserve a separate study that goes beyond the scope of this paper.*

My final comment goes to the core of the matter: should the propagation of TEs via drift in small subpopulations and the causal role of TEs in speciation be viewed as mutually exclusive? Is a feedback imaginable whereby TEs propagate by drift and then contribute to reproductive isolation?

**Response**: *Excellent point. We added a new section "Carrier subpopulations and the origin of species" to outline the historical perspective and conditions when the role of TEs in speciation could be of particular importance. We also discuss this point under implications of the hypothesis.*

Koonin, E. V. (2009) "Intron-Dominated Genomes of Early Ancestors of Eukaryotes." *J Hered *100: 618-623.

Lynch, M. (2007) "The Frailty of Adaptive Hypotheses for the Origins of Organismal Complexity." *Proc Natl Acad Sci USA *104 Suppl 1: 8597-8604.

Lynch, M. (2007). *The Origins of Genome Archiecture*. Sunderland, MA: Sinauer Associates.

### Reviewer 2

Jürgen Brosius, University of Muenster, Germany

This is an interesting, but highly speculative manuscript on the connection of transposable elements (TEs) to speciation in the light of population genetics, termed the carrier subpopulation (CASP) hypothesis.

Common sense would dictate that fixation of TEs is associated with speciation and that occasionally TEs facilitate or even trigger speciation. Just as most TE insertions are neutral to slightly deleterious, occasionally are selected against and rarely result in exaptation of a novel functional part of a gene. Hence, the two views should not be presented as being diametrically opposed. Furthermore, not only fixation but also the activity of retroposition should be taken into consideration. For example, in case of non-autonomous TEs, the expression and structure of the RNA template or the efficiency of the machinery harbored by a simultaneously active autonomous element and their interplay determines burst or bust.

**Response**: *As stated in the paper, TEs can affect speciation but their amplification in small populations is a more general mechanistic phenomenon, which we propose to use as a "null hypothesis" for analysis of the undeniable and diverse impact of TEs (see the response to Reviewer 1). See section: "Carrier subpopulations and the origin of species". In response to the second point, it is emphasized in the "Presentation of the hypothesis" section that the rate of (retro)transposition is the same as the fixation rate under relaxed selection in small populations.*

### Major points

1) The presence of many (young) retroposons in the various species crucially depends on the state of annotation (high, for example in pica; low in rabbit and hare). An additional explanation for species with the highest numbers of young families in Table 2 such as mouse (23 families), rat (21), bat (15), Rhesus macaque (15) and human (12), representing the largest mammalian orders of Rodentia, Chiroptera and Primates could be a faster clock (higher mutation rate) compared to other mammals as sure is the case in rodents.

**Response**: *Annotation is particularly problematic for older families because they are often difficult to define due to high sequence divergence. Therefore, we focused on relatively young families. These were studied for quite some time before the table was generated. Regarding the mutation rate: there is more TE activity in humans than in chimpanzees (see Table *2*and Britten, R.J. PNAS, 107: 19945-19948). It is debatable whether or not humans mutate faster than chimpanzees. Furthermore, a faster clock means faster elimination of TEs. For example, MIRs are much less preserved in rodents than in humans. The CASP hypothesis links the activity of TEs to population structure and genetic drift. Certainly, there are complicated variations on the theme when selection is considered, but selection models are beyond the scope of the hypothesis and of this paper.*

2) A major problem is that the data in Table 2 upon which the hypothesis is based on are incomplete if not selected.

There are large numbers of TEs or TE families in Xenarthra and Afroteria some of them highly specific to genera and species (for examples see: [Churakov G, Smit AF, Brosius J, Schmitz J (2005) A novel abundant family of retroposed elements (DAS-SINEs) in the nine-banded armadillo (Dasypus novemcinctus). Mol Biol Evol. 22:886-93; Möller-Krull M, Delsuc F, Churakov G, Marker C, Superina M, Brosius J, Douzery EJ, Schmitz J (2007) Retroposed elements and their flanking regions resolve the evolutionary history of xenarthran mammals (armadillos, anteaters, and sloths). Mol Biol Evol 24:2573-82; in particular see Figure 3]; Nishihara H, Kuno S, Nikaido M, Okada N. MyrSINEs: a novel SINE family in the anteater genomes. Gene. (2007) 400:98-103; Nishihara H, Satta Y, Nikaido M, Thewissen JG, Stanhope MJ, Okada N (2005) A retroposon analysis of Afrotherian phylogeny. Mol Biol Evol 22:1823-33.]

In the Platypus genome [Warren WC et al. (2008) Genome analysis of the platypus reveals unique signatures of evolution. Nature 453:175-83] there are more than 40 specific families of L2 LINEs and associated Mon-SINEs. These families are not present in other sequenced genomes of birds and mammals. In several Mon-SINE families there is less than 2% divergency, which would indicate young age (Supplementary Notes S20 in the aforementioned citation).

**Response**: *Many families of TEs from the species listed above were discovered and entered to Repbase by the authors of this manuscript. Nevertheless, most of them are more than 5% divergent from their consensus sequences (even though they are species- or lineage-specific), and we decided not to detail them in this hypothesis. They deserve a separate in-depth analysis and we stand by this decision. However, we gratefully acknowledge a large underlying point made by the reviewer: we did not take advantage of a large body of excellent phylogenetic studies involving old families of TEs, due to the original focus on response to the "genomic drive hypothesis". This omission is remedied and phylogenetic analyses are prominently cited in the revised manuscript.*

3) CASP also proposes that small subpopulations resulting from the subdivisions are primary incubators of new repetitive families originally fixed by genetic drift, and subsequently undergoing purifying selection. I doubt that most new repetitive families are under purifying selection. There are a few exceptions where novel RNAs, namely BC1 and BC200 RNA, itself generated by retroposition in rodents and primates, respectively, were exapted into a function. Now, they are under purifying selection and also happened to be master genes for repeats (a subfamily of ID SINEs and BC200-derived monomeric Alus, respectively).

**Response**: *This must be a misunderstanding since we certainly did not mean to imply that "most new repetitive families are under purifying selection." However, a certain fraction of elements will eventually become a conserved part of the genome. We rephrased the sentence and included ref. 83 to broaden the context.*

4) "However, the lack of young families in genome is less informative because it may be caused by random loss of active source genes in founder populations."

In contrast to the mosquito genome, the honeybee (Apis mellifera) has no active TEs except for two families of DNA transposons [Weinstock et al (2006) Insights into social insects from the genome of the honeybee Apis mellifera. Nature 443: 931-949]. There are 32 known Apis species, 24 of which are close to Apis mellifera. This would contradict CASP. Perhaps CASP does not apply to all phylogenetic branches?

Another observation is the paucity of TEs in birds despite a tremendous speciation activity during neoavian radiation [Suh, A., Paus, M., Kiefmann, M., Churakov, G., Franke, F.A., Brosius, J., Kriegs, J.O., Schmitz, J. (2011) Mesozoic retroposons reveal parrots as the closest living relatives of passerine bird. Nature Communications, in press]. An additional problem is that one cannot conclude from extant species to species diversity in the various lineages over the past dozens of million years.

**Response**: *The CASP hypothesis predicts that at least some species including those living in marginal niches (e.g. primitive plants), or species such as the honeybee do not encounter enough opportunities to explore new alternative niches and to split into many small subpopulations for any extended period of time. The same may apply to "fine-tuned" parasites and deserves a separate analysis. On the other side of the spectrum are birds that can disperse more readily than mammals into local subpopulations (with the possible exception of bats). Therefore, in birds high activity of TEs might have been less relevant for speciation. According to the hypothesis, mammalian populations certainly preserved many smaller bird-like families of TEs, but they are not necessarily associated with speciation. For example, there are multiple families of LTR retrotransposons in mammals, but they probably didn't proliferate fast enough to sufficiently affect the divergence of different subpopulations, which is a prerequisite for the origin of new species with large families.*

### Minor points

1) The manuscript would gain a lot by including more references.

Just to give a few examples, such as earlier publications on the role of TEs in speciation: [Bingham PM, Kidwell MG, Rubin GM (1982) The molecular basis of P-M hybrid dysgenesis: the role of the P element, a P-strain-specific transposon family. Cell. 1982 Jul;29(3):995-1004]. Or, publications on the source, founder, master gene concepts: [Shen MR, Batzer MA, Deininger PL (1991) Evolution of the master Alu gene(s). J Mol Evol33:311-20]. Or, a reference concerning the proposal that Alu elements were probably removed from the male sex chromosome over time (page 10).

In support to some suggestions, fixation of individual TEs in connection to the population structure was proposed based on experimental data in the following articles: [Churakov G, Kriegs JO, Baertsch R, Zemann A, Brosius J, Schmitz J (2009) Mosaic retroposon insertion patterns in placental mammals. Genome Res19:868-75; Nishihara H, Maruyama S, Okada N (2009) Retroposon analysis and recent geological data suggest near-simultaneous divergence of the three superorders of mammals. Proc Natl Acad Sci USA 106:5235-40; Churakov G, Sadasivuni MK, Rosenbloom KR, Huchon D, Brosius J, Schmitz J (2010), Rodent evolution: back to the root. Mol Biol Evol 27(:1315-26]. These findings should be discussed in connection to CASP hypothesis.

**Response**: *All the references under this point and many listed above are added and discussed in the context of the revised manuscript.*

2) The TINE1 element is not a good example to illustrate a "new family" of retroelements. TINE1 is not specific for the tarsier family as it is found in other primates, and hence a "master gene" appeared prior to tarsier speciation. In addition, characterization of TINE1 as a "processed pseudogene" is questionable, because there is no processing except polyadenylation at a solitary LTR.

**Response**: *Deleted.*

3) The sentence: " The hypothesis predicts that repetitive families represent only the most visible and probably most abundant portion of all types of mutations be fixed in small populations in addition to TEs." is unclear: Repetitive families consist of TEs?

**Response**: *Rephrased and clarified. Throughout the paper "repetitive families" and "families of TEs" are used interchangeably.*

4) Not clear why the AluYa5 and AluYb8 families of repeats are of particular interest? Why are the ratios of chromosomal densities of AluYa5 elements consistent with the model of paternal transmission, i.e. inheritance of active Alu elements through male germ line only?

**Response**: *These families are the largest among the potentially active ones and as such they are subject to meaningful statistics. The second point: apparently Alu elements are activated in male germ line. This may be related to de-methylation of chromosome Y (see reference 37 in publication 61 cited in this paper).*

5) "... that Alu elements were probably removed from the male sex chromosome over time." - citation is necessary, and explanation of the removal process would be useful.

**Response**: *See ref. 61 page 1271 (bottom) and page 1272 (top).*

6) CSINE2 - is a family present in pika and all Leporidae and were active before pika and rabbit speciation [Kriegs JO, Zemann A, Churakov G, Matzke A, Ohme M, Zischler H, Brosius J, Kryger U, Schmitz J (2010) Retroposon insertions provide insights into deep lagomorph evolution. Mol Biol Evol 27:2678-81, Figure 2] and are present in pika and rabbit genomes in comparable amounts (see: previous citation, Figure 1). It is too speculative to discuss divergence of CINE2 in rabbit genome without comparison with pika genomic data.

**Response**: *We added Figure *3*with cumulative numbers of all families and for phylogenetic details we included the reference to Kriegs et al. (70).*

## Reviewer 3

I. King Jordan, Georgia Institute of Technology, USA

In this compelling hypothesis paper, Jerzy Jurka and colleagues lay out their vision for the relationship between the genome dynamics of transposable elements (TEs) and the process of speciation. They present the 'carrier subpopulation (CASP)' hypothesis, which emphasizes that species-specific differences in TE family composition are best explained by differences in species' population structure. The basic idea of the CASP hypothesis is that subdivided populations will inherit distinct sets of active TEs, or TE subfamilies, and furthermore different subsets of these TEs will be randomly fixed by genetic drift among the divided subpopulations. Meanwhile, given sufficient time and the availability of distinct niches, the divided subfamilies will diverge into new species. Together, this will lead to a greater relative diversity of young TE families for lineages that include numerous species. The CASP hypothesis is notable for the fact that it posits a passive, rather than a causal, role for TE activity and accumulation in the process of speciation. As such, the CASP hypothesis serves as a counter-point to the 'genomic drive' hypothesis for the significance of TEs with respect to speciation, which holds that amplification of TEs leads to speciation by increasing genomic variability.

The presentation of the CASP hypothesis in this paper is interesting, thoughtful and timely, and I expect that this work will be quite thought provoking to investigators working on TEs, evolution and genomics. Thus, I certainly support publication of the work in Biology Direct. Below, I provide a number of comments, questions and suggestions that the authors may wish to consider prior to finalization of their manuscript.

1. There are assumptions of the CASP model that need to be critically interrogated. One critical assumption of the model is that fixation of repetitive families takes place primarily in small populations by genetic drift. Of course, it is entirely reasonable to posit that random fixation any genetic element would occur preferentially in small populations. However, this assumption seems to imply that TE fixation dynamics are dominated, or even exclusively shaped, by population level forces. In fact, the population dynamics of repetitive elements can be considered to take place at two levels - there are indeed population level dynamics predicated upon differential reproductive success of individual organisms but there are also genome level dynamics based upon differential reproductive success of individual TE copies or subfamilies. This understanding was articulated in the early formulation of the selfish DNA theory when it was theoretically demonstrated that TEs could increase in copy number even if their replication was deleterious to the host (Hickey 1982 Genetics 101: 519). In other words, the genome level replication dynamics of TEs could overcome the population level effects of selection. This may be an extreme view, but there is certainly an interplay between population dynamics and genome level dynamics when it comes to TE replication and fixation. Any model that only treats one or the other of these two important levels may be missing a critical component.

**Response**: *The CASP hypothesis is based on neutral theories (refs. 27-29) that include slightly deleterious mutations under relaxed selection in small populations. Under relaxed selection the mutation rate by TEs (the rate of transposition) and the fixation rate are the same, which may leave the impression that one is missing.*

2. Another important aspect of the model is the idea that randomly divided subpopulations will inherit different sets of active TE copies (Figure 1). On its face this seems quite reasonable. However, the master copy model for TE replication holds that one or a few copies of a TE (sub)family are primarily responsible for the replication and ongoing expansion of the family. If there are only a few master copies, or if they are highly identical in terms of sequence/structure, then the likelihood of different subpopulations inheriting distinct sets of active TEs would seem to be reduced. How well does the master copy model hold for the species examined here, in particular for the primate lineage with respect to Alus and L1s, which are discussed at length? And how would this impact the CASP hypothesis?

**Response**: *The CASP hypothesis permits subpopulations without active TEs. However, such subpopulations may be less likely to diversify fast enough to become foundations for new species (see section "Carrier subpopulations and the origin of species"). The hypothesis implies that very active TEs are most likely to contribute to productive speciation. Nevertheless, they can be very destructive and only a few of them left behind viable subpopulations that were foundations for new species. They also left behind large repetitive families in primates that were the basic evidence for the master gene hypothesis (refs. 10-15). Slowly replicating TEs are much less likely to affect speciation. They are also less destructive and probably less frequently suppressed by the silencing mechanisms. Therefore, they are more likely to be represented by multiple active copies as proposed by the "transposon model" (refs. 47-48).*

3. There is one slightly troubling (or perhaps simply confusing) aspect of the CASP hypothesis articulated in the conclusion (implications) section of the article. Here, the authors mention that the 'genomic record of young TEs can be a powerful indicator of... subdivisions in the population that underlie speciation events' but then go on to state that the opposite pattern of a lack of young TE families in a genome may not be informative because it could be due to other factors. This would seem to suggest that the hypothesis lacks discriminating power with respect to the relationship between the extent of young TE families in a genome and speciation rates along a lineage. Is this really the case? Does this mean that one would not be able to systematically relate the extent, or lack, of young TE families to high or low levels of speciation?

**Response**: *The CASP hypothesis links speciation to population subdivision, which is driven by the availability of biological niches. There are three basic categories of subpopulations: (1) those that do not carry any active TEs but still evolve into new species, due to geographical factors allowing both survival and reproductive isolation; (2) subpopulations that carry moderately active TEs that have little or no impact on potential speciation events and (3) subpopulations that are rapidly mutated by very active TEs and, if they survive, they are likely to become founding populations for new species. Given the ubiquitous nature of TEs, the first category of subpopulations is probably rare. We think that the most common is category 2 and majority of the diverse families of TEs originate in such subpopulations (see "Origin of diverse families of TEs"). The last category is the "extreme version" of the category 2.*

4. Michael Lynch has written extensively on the importance of non-adaptive aspects of evolution, *i.e*. the role of genetic drift, in shaping genome architecture (see his book The Origins of Genome Architecture). Similar to what is proposed here, Lynch holds that TEs are able to accumulate to high copy numbers owing to the reduced efficacy of natural selection in small populations. The CASP hypothesis should be considered in light of the previous work of Lynch along with the ensuing discussion (controversy) that his work engendered.

**Response**: *In the last section we discuss the relationship between the CASP hypothesis and the Lynch & Conery hypothesis in the context of the ongoing debate on the role of drift in evolution of genomic complexity.*

5. The authors make a very clear and strong statement in the abstract regarding the relationship between the numbers of young TEs in genomes and the number of species in a lineage. In the body of the manuscript, they go on to provide data on the diversity of TE families among several vertebrate, plant and insect genomes (Table 1) and additional more detailed data on the diversity of TEs in mammalian species (Table 2). However, it was not immediately apparent how, or even whether, these data directly support the CASP hypothesis. For example, they show that closely related Anopheles species have large differences in TE diversity and they speculate as to the pattern of population subdivision this would predict, but they do not confirm whether this conjecture is borne out by the data. Similarly for Table 2, the authors discuss the data in depth as they relate to various aspects of TE and species population dynamics, but they don't show a clear pattern of high numbers of young TEs and high numbers of species in a lineage. It would really help the reader to clearly and succinctly point out how the TE diversity data do or do not support the hypothesis. More to the point, a clear and quantitative (perhaps a regression analysis?) demonstration of the relationship between the numbers of young TEs in genomes and the numbers of species in a lineage is needed to provide support for the unequivocal statement made in the abstract.

**Response**: *The main points can be summarized as follows (with some oversimplification): (1) Multiple families of TEs in a genome are associated with multiple subpopulations in the historical population from which the genome has emerged (younger multiple families are associated with more recent subdivisions). (2) Speciation events are likely to correlate with the cumulative number of subpopulations that originated (and vanished) during the history of a lineage and indirectly with the number of families of TEs generated in those subpopulations (recent speciation events are likely to correlate indirectly with recent fixations of repetitive families). (3) On average, there should be higher proportions of surviving species from recent speciation events than from the old ones.*

*Currently, there is not enough data to correlate the number of species or speciation events with the population structure over geologic time. However, we found a way to indirectly support the first point above by showing a significant positive correlation between two unrelated families (see Figure *2*) based on the prediction that they both correlate with the number of subpopulations in a population.*

6. A corollary to comment #5 is that the best hypotheses make very specific predictions that can be empirically tested - or in the case of evolutionary hypotheses at least interrogated via direct observations on standing variation. The CASP hypothesis directly contradicts the genomic drive hypothesis (Oliver and Greene 2009 Bioessays 31:703) with respect to the agency of TEs in the process of speciation. Here, it would help if the authors could set up some mutually exclusive predictions that would clearly distinguish these two hypotheses. Oliver and Greene have recently published additional evidence for their genomic drive hypothesis (Oliver and Greene 2011 Mobile DNA 2: 8). Consideration of this work could be relevant to the authors' efforts to distinguish the two hypotheses.

**Response**: *The basic premise of the genomic drive hypothesis is that TEs constitute the main engine of the process that can result in the generation of "widely divergent new taxa, fecund lineages, lineage selection, and punctuated equilibrium." We find this premise untenable without rooting it in a broader context of the population structure, which is the frame of reference for the definition of species and other taxonomic units in a lineage (see ref. 81). Furthermore, in their papers Oliver and Greene ignored the fundamental problem of reproductive isolation. In the most recent version of the hypothesis published in Mobile DNA, the authors moved closer to the population genetics perspective by invoking "environmental and ecological factors." However, they ended up with enumeration of changes introduced by TEs in primates to support their premise. Therefore, we find little theoretical overlap between the CASP hypothesis based on the fundamental concepts of the population genetics and the genomic drive hypothesis except that both hypotheses attempt to make sense of the undeniable evidence linking TEs and speciation. In the revised manuscript we comment only on the concept of "fecundity" in the context of large mammalian orders and briefly address the main premise of the hypothesis in the Implications section.*

7. The authors work at the Genetic Information Research Institute - the home of Repbase - and thus would seem to have a uniquely close perspective and deep insight into the distribution of TE diversity among evolutionary lineages. However, the rationale behind the choice of species/lineages represented among the primary data presented in Tables 1 &2 may not be immediately apparent to readers less familiar with Repbase. It would help to have a clear explanation for how and why the genomes and lineages represented among these data were chosen.

**Response**: *First, we focused on young families of TEs to minimize uncertainties associated with annotations of older repeats. In *Table 1*we tried to focus on well annotated species to illustrate the proposed correlation between population structure and the diversity of TEs. In *Figure 2, *inspired by this review, we use a more extensive dataset from Repbase to verify the predicted correlation. In *Table 2*we focus on mammalian species that are not only well annotated in Repbase, but they also have been extensively studied from the evolutionary point of view. This helped to combine multiple lines of published evolutionary evidence in support of the hypothesis.*
